# Supplementary material for: From Pathways to Practice: Impact of Implementing Mobilization Recommendations in Head and Neck Cancer Surgery with Free Flap Reconstruction
Source: Cancers (Basel). 2021 Jun 9;13(12):2890. doi: 10.3390/cancers13122890 (PMC8228478; doi:10.3390/cancers13122890)
Supplement: Supplementary file 1 [file cancers-13-02890-s001.zip › cancers-1213921-supplementary.pdf]

Table S1. Improved compliance and time to postoperative mobilization with the recommendation after the mobilization recommendation was introduced. Fisher's exact tests were used for *p*-values, except for mobilization (*Mann Whitney Test).

	All cases *n*=445	Before *n*=164	After *n*=281	Odds ratio (95% CI)	*p*-value
**Compliance**					
Yes	267 (60)	88 (54)	179 (64)	1.52 (1.00-2.28)	**0.045**
No	178 (40)	76 (46)	102 (36)		
**Mobilized within 24 hours**					
Yes	196 (44)	17 (10)	179 (64)	15.2 (8.5-28.4)	**<0.001**
No	249 (56)	147 (90)	102 (36)		
**Mobilized within 48 hours**					
Yes	342 (77)	88 (54)	254 (90)	8.1 (4.8-13.9)	**<0.001**
No	103 (23)	76 (46)	27 (10)		
**Mobilized within 72 hours**					
Yes	405 (91)	136 (83)	269 (96)	4.6 (2.2-10.3)	**<0.001**
No	40 (9)	28 (17)	12 (4)		
**Time to mobilization**					
Mean ± SD	2.2 ± 2.5	2.9 ± 2.0	1.7 ± 2.6		
Median (IQR)	2 (1-2)	2 (2-3)	1 (1-2)	$z = 11.48$	**<0.001***

Table S2. Logistic regression analysis (controlling for alcohol) of compliance with guideline concordant care, postoperative complications, and length of stay. The reference level for odds ratios is indicated with *italics*.

Characteristic	All cases	Compliance=Yes	Compliance=No	OR	95% CI	*p*-value
n (%)	445 (100)	267 (60)	178 (40)			
**Major complication**						
Yes	70 (15.8)	29 (10.9)	39 (21.9)	0.44	0.25-0.76	**0.004**
No	374 (84.2)	238 (89.1)	123 (69.1)			
**Pneumonia**						
Yes	37 (8.3)	15 (5.6)	22 ()	0.35	0.16-0.75	**0.007**
No	408 (91.7)	252 (94.4)	156 ()			
**Length of stay**						
POD 0-10	204 (46)	131 (49.1)	109 (61.6)	0.58	0.38-0.59	**0.013**
After POD 10	240 (54)	136 (50.9)	68 (38.4)			

Table S3. Predictors of major complications using elastic net regularization followed by multivariable logistic regression. Mobilization (within 48 hours) substituted for compliance with guideline-recommended mobilization.

**Characteristic** *(reference level)*	**Major complications**			
	**Odds ratio**	**95% CI**	***z***	***p*-value**
**Age** (centred at the mean)	1.01	0.98-1.04	0.56	0.558
**BMI category** *(healthy range)*				
Underweight	1.93	0.57-6.54	1.05	0.293
Overweight	0.44	0.19-1.02	-1.91	0.056
Obese	0.74	0.32-1.73	-0.69	0.493
**Sex** *(male)*				
Female	0.70	0.33-1.50	-0.91	0.362
**Smoking Status** *(never)*				
Former smoker	0.57	0.24-1.35	-1.28	0.201
Current smoker	0.23	0.08-0.65	-2.74	**0.006**
**Alcohol status** *(never)*				
Light/Moderate	1.86	0.75-4.61	-1.34	0.181
Heavy	1.32	0.40-4.32	0.46	0.645
Former drinker	0.52	0.12-2.27	-0.87	0.383
**Comorbidity** *(none)*				
One	1.41	0.57-3.49	0.74	0.460
Two or more	2.64	1.14-6.15	2.25	**0.024**
**Primary Site (***oral cavity)*				
Pharynx & Larynx	1.42	0.49-4.07	0.65	0.516
Paranasal/Nasal	0.31	0.05-1.81	-1.30	0.192
Skin	0.17	0.01-1.90	-1.44	0.150
Other	0.32	0.05-1.92	-1.25	0.212
**Cancer stage** *(I-II)*				
III-IV	2.29	0.96-5.48	1.86	0.063
**Flap type***(radial forearm)*				
Fibula	1.28	0.52-3.14	0.53	0.597
Anterolateral thigh	1.39	0.45-4.29	0.57	0.566
Other	2.80	1.12-7.01	2.20	**0.028**
**Unit arrival** *(POD 0-1)*				
After POD 1	2.62	1.17-5.87	2.33	**0.020**
**Compliance** *(no)*				
Yes	0.57	0.29-1.13	-1.62	**0.106**
**Tracheostomy** *(no)*				
Yes	0.38	1.13-1.07	-1.83	**0.067**

Table S4. Predictors of hospital length of stay using elastic net regularization followed by multivariable logistic regression. Mobilization (within 48 hours) substituted for compliance with guideline-recommended mobilization.

**Characteristic** *(reference level)*	**Major complications**			
	**Odds ratio**	**95% CI**	*z*	***p*-value**
**Age** (centred at the mean)	1.03	1.01-1.05	2.43	**0.015**
**BMI category** *(healthy range)*				
Underweight	1.00	0.34-2.96	-0.01	0.994
Overweight	0.64	0.34-1.20	-1.39	0.165
Obese	0.48	0.23-0.98	-2.02	**0.043**
**Alcohol status** *(never)*				
Light/Moderate	0.58	0.29-1.17	-1.51	0.130
Heavy	0.80	0.35-1.78	-0.56	0.577
Former drinker	2.37	0.90-6.21	1.75	0.081
**Comorbidity** *(none)*				
One	1.00	0.51-1.96	0.00	0.998
Two or more	1.50	0.75-3.01	1.14	0.253
**Primary Site (***oral cavity)*				
Pharynx & Larynx	4.69	1.65-13.35	2.90	**0.004**
Paranasal/Nasal	0.06	0.01-0.33	-3.26	**0.001**
Skin	0.12	0.02-0.54	-2.75	**0.006**
Other	0.73	0.19-2.76	-0.46	0.646
**Flap count** *(one)*				
Two	0.21	0.01-6.44	-0.89	0.375
**Resection extent** *(soft tissue only)*				
Bone	1.15	0.60-2.20	0.43	0.669
Soft tissue & bone	27.10	0.49-1493.71	1.61	0.107
**Compliance** *(no)*				
Yes	0.89	0.51-1.55	-0.41	**0.680**
**Tracheostomy** *(no)*				
Yes	3.03	1.21-7.60	2.36	**0.018**
**Any complication** *(no)*				
Yes	6.69	3.75-11.95	6.42	**<0.001**
**Major Complication** *(no)*				
Yes	2.29	0.91-5.76	1.76	0.079
